# ^18^F-FDG PET/CT may be a suitable method for preoperative diagnosis and evaluation of Chinese older patients with hilar cholangiocarcinoma

**DOI:** 10.1186/s12877-018-0846-8

**Published:** 2018-07-06

**Authors:** Xuling Li, Ying Zhang, Yongyang Zhang

**Affiliations:** 1 Department of Pharmacy, Jining No.1 People’s Hospital, Jining, China; 2 Department of Surgery, Guangzhou Eighth People’s Hospital, Guangzhou Medical University, Guangzhou, China; 3 Emergency Department, Jining No.1 People’s Hospital, Jining, China

**Keywords:** Chinese older patients, ^18^F-FDG PET/CT, Hilar cholangiocarcinoma, Preoperative evaluation

## Abstract

**Background:**

As the most common cholangiocarcinoma, hilar cholangiocarcinoma (HCCA) is a challenge in hepatobiliary surgery and causes a very poor prognosis. This study was designed to explore whether ^18^F-fluorodeoxyglucose positron emission tomography/computed tomography (^18^F-FDG PET/CT) may be a suitable method for preoperative diagnosis and evaluation of Chinese older patients with hilar cholangiocarcinoma.

**Methods:**

This study enrolled 53 patients (≥ 65 years) with HCCA. ^18^F-FDG PET/CT scan was performed in all patients within one week before operation.

**Results:**

^18^F-FDG PET/CT identified the tumors in all patients (100%). There were 48 patients (90.6%) with the same Bismuth-Corlette classifications determined by ^18^F-FDG PET/CT and operative pathology, whereas Bismuth-Corlette classifications of 5 patients (9.4%) were underestimated by ^18^F-FDG PET/CT compared with that determined by operative pathology. ^18^F-FDG PET/CT identified 19 patients (sensitivity: 67.9%) in 28 patients with lymph node metastases, and 22 patients (specificity: 88.0%) in 25 patients without lymph node metastases, with an accuracy of 77.4%. ^18^F-FDG PET/CT identified 8 patients (sensitivity: 47.1%) in 17 patients with liver, peritoneal or other distant metastases, and 35 patients (specificity: 97.2%) in 36 patients without liver, peritoneal or other distant metastases, with an accuracy of 81.1%. ^18^F-FDG PET/CT identified 17 patients (sensitivity: 73.9%) in 23 patients with unresectable tumors, and 24 patients (specificity: 80.0%) in 30 patients with resectable tumors, with an accuracy of 77.4%.

**Conclusions:**

^18^F-FDG PET/CT may be a suitable method for preoperative diagnosis and evaluation, and offer valuable information for effective operation in Chinese older patients with HCCA.

## Background

As the most common cholangiocarcinoma, hilar cholangiocarcinoma (HCCA) involves common hepatic duct, bifurcation of common hepatic duct, and left and right hepatic ducts [1]. HCCA is a challenge in hepatobiliary surgery, and often has unsatisfactory operative results [2]. In China, its incidence has been growing year by year, and accounts for 40–60% of cholangiocarcinom with an incidence of 0.01–0.20% in postmortem examination [3]. It occurs more commonly in older patients, and causes a very poor prognosis [4]. Older patients with HCCA have no only worse general condition and more prevalent co-morbidity, but also more complex anatomical structure and metabolic status [4]. Thus, HCCA is more difficult to be diagnosed and removed in older patients [5]. Preoperative diagnosis and evaluation can play crucial roles in improving operative success rate and five-year survival rate in older patients with HCCA [6]. Although imaging methods, such as abdominal ultrasound, computed tomography and magnetic resonance imaging, have noninvasive advantage, they do not fully meet the need of preoperative diagnosis and evaluation in older patients with HCCA [7].

^18^F-fluorodeoxyglucose positron emission tomography (^18^F-FDG PET) has been valuable in providing significant tumor-related metabolic information that is critical to diagnosis [8]. ^18^F-FDG PET/computed tomography (CT) is a unique combination of the cross-sectional anatomic information provided by CT and the metabolic information provided by PET, which are acquired during a single examination and fused [8]. As a non-invasive method simultaneously offering anatomical and functional images, ^18^F-FDG PET/CT has the potential to achieve preoperative diagnosis and evaluation of older patients with HCCA [9]. However, there are limited studies analyzing these roles of ^18^F-FDG PET/CT in Chinese older patients with HCCA [10]. Aim of the present study was to explore whether ^18^F-FDG PET/CT may be a suitable method for preoperative diagnosis and evaluation of Chinese older patients with HCCA.

## Methods

### Patients

The present study enrolled 53 patients (≥ 65 years) with HCCA in Jining No.1 People’s Hospital from December 2011 to May 2017. HCCA was diagnosed by clinical appearance and operative pathology. ^18^F-FDG PET/CT scan was performed in all patients within one week before operation.

### Pet/CT

Patients were injected with ^18^F-FDG (5.55 MBq/kg) and scanned by Siemens Biograph 64 high definition PET/CT. Scanned area ranged from the skull base to the upper femur. All images were reconstructed by Ordered Subset Expectation Maximization, and analyzed by radiologist with full experience. Standardized Uptake Value maximum (SUVmax) levels were measured from the region of interest in ^18^F-FDG PET/CT image, and local SUVmax levels × 2.5 were applied to decide the local radioactive concentration [8]. ^18^F-FDG PET/CT identified primary tumor, lymph node and distant metastases based on the local radioactive concentration, and operative pathology determined these lesions as the gold criteria [8].

### Criteria

Tumors were divided into four types with Bismuth-Corlette classifications: 1) type I involved common hepatic duct but not its bifurcation; 2) type II involved common hepatic duct and its bifurcation but not left and right hepatic ducts; 3) type III involved common hepatic duct and its bifurcation, and right hepatic duct (IIIa) or left hepatic duct (IIIb); 4) type IV involved common hepatic duct and its bifurcation, and right and left hepatic ducts [11]. Based on Burke criteria, patients with unresectable tumors were further identified in addition to those without ability to tolerate a major operative procedure: 1) secondary hepatic ducts bilaterally involved; 2) main trunk or bifurcation of portal vein or hepatic artery involved; 3) liver parenchyma widely involved in hepatic hilar region or two liver lobes; 4) lymph node metastases outside hepatic pedicle; 5) liver, peritoneal or other distant metastases [12].

### Statistics

Continuous variable was described as mean and standard deviation (normal distribution) or median and range (skewed distribution), whereas categorical variable was described as number and percentage. Sensitivity and specificity are generally accepted statistical method in diagnostic test, and widely applied in the present study [13]. Statistics were conducted by Statistical Package for Social Sciences 17.0 software (Chicago, IL., USA).

## Results

Study patients were 68 (66–77) years of age, and there were 36 males (67.9%). Demographic information, clinical symptoms, TNM stages and operative pathology of all patients are shown in Table 1. ^18^F-FDG PET/CT identified the tumors in all patients (100%). There were 48 patients (90.6%) with the same Bismuth-Corlette classifications determined by ^18^F-FDG PET/CT and operative pathology, whereas Bismuth-Corlette classifications of 5 patients (9.4%) were underestimated by ^18^F-FDG PET/CT compared with that determined by operative pathology (Table 2). ^18^F-FDG PET/CT identified 19 patients (sensitivity: 67.9%) in 28 patients with lymph node metastases, and 22 patients (specificity: 88.0%) in 25 patients without lymph node metastases, with an accuracy of 77.4% (Table 3). ^18^F-FDG PET/CT identified 8 patients (sensitivity: 47.1%) in 17 patients with liver, peritoneal or other distant metastases, and 35 patients (specificity: 97.2%) in 36 patients without liver, peritoneal or other distant metastases, with an accuracy of 81.1%. ^18^F-FDG PET/CT identified 17 patients (sensitivity: 73.9%) in 23 patients with unresectable tumors, and 24 patients (specificity: 80.0%) in 30 patients with resectable tumors, with an accuracy of 77.4%.

**Table 1:** Characteristics of all patients

Characteristics	Patients (*n* = 53)
Demographic information	
Age	68 (66–77)
Male	36(67.9%)
BMI	22.7(19.0–25.6)
Clinical symptoms	
Jaundice	36(67.9)
Stomachache	24(45.3)
Skin itch	22(41.5)
Nausea	27(50.9)
TNM stages	
Type I	11(20.8%)
Type II	14(26.4%)
Type III	5(9.4)
Type IVa	6(11.3)
Type IVb	17(32.1)
Operative pathology	
Lymph node metastases	28(52.8)
Distant metastases	17(32.1)
Unresectable tumors	23(43.4)

*BMI* Body mass index

**Table 2:** Bismuth-Corlette classifications using ^18^F-FDG PET/CT compared with operative pathology

Operative pathology	^18^F-FDG PET/CT					
	Type I	Type II	Type IIIa	Type IIIb	Type IV	Total
Type I	7	0	0	0	0	7
Type II	0	6	0	0	0	6
Type IIIa	0	1	8	0	0	9
Type IIIb	0	2	0	12	0	14
Type IV	0	0	1	1	15	17
Total	7	9	9	13	15	53

^18^*F-FDG PET/CT* ^18^F-fluorodeoxyglucose positron emission tomography/computed tomography

**Table 3:** Preoperative evaluation using ^18^F-FDG PET/CT compared with operative pathology

Operative pathology	Status	^18^F-FDG PET/CT		Sensitivity	Specificity	Accuracy
		Yes	No			
Lymph node metastases	Yes	19	9	67.9%	88.0%	77.4%
	No	3	22			
Distant metastases	Yes	8	9	47.1%	97.2%	81.1%
	No	1	35			
Unresectable tumors	Yes	17	6	73.9%	80.0%	77.4%
	No	6	24			

^18^*F-FDG PET/CT* ^18^F-fluorodeoxyglucose positron emission tomography/computed tomography

## Discussion

Radiotherapy and chemotherapy have no definite effect on HCCA, and operative therapy is a major method for patients with HCCA [14]. However, as a challenge faced by hepatobiliary surgeons, HCCA surgery is difficult to be operated and successful, and easy to cause intractable complications and poor prognoses [15]. Making definite diagnosis, judging tumor classifications and detecting different metastases are crucial for operative success, and identifying unresectable tumors can avoid unnecessary operation [16]. Compared with abdominal ultrasound, computed tomography and magnetic resonance imaging, ^18^F-FDG PET/CT shows possible superiority in preoperative diagnosis and evaluation for patients with HCCA due to the combination of functional and anatomical images [9]. With rapid growth of malignant cells and obvious increase of anaerobic glycolysis, there are more uptake and storage of ^18^F-FDG in HCCA cells than other cells, making the superiority of ^18^F-FDG PET/CT possible [9]. However, limited studies have observed the roles of ^18^F-FDG PET/CT in making definite diagnosis, judging tumor classifications, detecting different metastases and identifying unresectable tumors of Chinese older patients with HCCA [10].

HCCA is more commonly developed in older patients, and more difficultly diagnosed in older patients [5]. Firstly, general condition is worse and co-morbidity is more prevalent in older patients with HCCA [4]. Secondly, anatomical structure and metabolic status were more complicated in these patients [4]. Preoperative diagnosis and evaluation can play crucial roles in improving operative success rate and five-year survival rate in older patients with HCCA [6]. However, previous studies have only analyzed one aspect of preoperative diagnosis and evaluation of patients with HCCA, such as resectable tumors. More importantly, these studies have not specially focused on older patients. The present study demonstrated that ^18^F-FDG PET/CT may be a suitable method for preoperative diagnosis and evaluation of Chinese older patients with HCCA. Sensitivity and specificity of computed tomography, magnetic resonance imaging and ^18^F-FDG PET/CT obtained by previous studies have been generally higher than the data from our study. There are obvious difficulties in preoperative diagnosis and evaluation of HCCA in order patients than in younger patients. Thus, computed tomography, magnetic resonance imaging and ^18^F-FDG PET/CT have reduced sensitivity and specificity in preoperative diagnosis and evaluation of HCCA.

Patients with HCCA at advanced stage always have very poor prognoses, and thus definite diagnosis before operation is of great implication [17]. In the present study, ^18^F-FDG PET/CT identified the tumors in all patients (100%), suggesting that ^18^F-FDG PET/CT may have suitable ability in making preoperative diagnosis of HCCA in Chinese older patients. Tumor classifications determine the operative method and success rate. Judging tumor classifications before operation can improve the accuracy of operative plan, quality of operative process and prognoses of operative patients [18]. There were 90.6% patients with Bismuth-Corlette classifications accurately determined by ^18^F-FDG PET/CT in the present study, suggesting that ^18^F-FDG PET/CT may have suitable ability in judging tumor classifications of HCCA in Chinese older patients.

Lymph node and distant metastases have crucial effects on the operative method and patient prognosis [19]. In addition to functional imaging, extensive inspection is another superiority of ^18^F-FDG PET/CT, which is suitable for identifying lymph node and distant metastases [8]. In the present study, ^18^F-FDG PET/CT identified lymph node metastases with sensitivity, specificity and accuracy of 67.9, 88.0 and 77.4%, and identified liver, peritoneal or other distant metastases with sensitivity, specificity and accuracy of 47.1, 97.2 and 81.1%, suggesting that ^18^F-FDG PET/CT may be useful in identifying lymph node and distant metastases of Chinese older patients with HCCA. Identifying unresectable tumors can reduce unnecessary operation and lighten patient suffering [6]. In the present study, ^18^F-FDG PET/CT identified unresectable tumors with sensitivity, specificity and accuracy of 73.9, 80.0 and 77.4%, suggesting that ^18^F-FDG PET/CT may offer valuable information in identifying unresectable tumors of Chinese older patients with HCCA.

## Conclusions

The present study demonstrated that ^18^F-FDG PET/CT may be a suitable method for preoperative diagnosis and evaluation, and offer valuable information for effective operation in Chinese older patients with HCCA. In the future, more studies are needed to confirm the conclusion in the present study.

## Abbreviations

^18^F-FDG PET/CT: ^18^F-fluorodeoxyglucose positron emission tomography/computed tomography
HCCA: Hilar cholangiocarcinoma
